# Age-related differences in the transient and steady state responses to different visual stimuli

**DOI:** 10.3389/fnagi.2022.1004188

**Published:** 2022-09-08

**Authors:** Xin Zhang, Yi Jiang, Wensheng Hou, Ning Jiang

**Affiliations:** ^1^Bioengineering College, Chongqing University, Chongqing, China; ^2^National Clinical Research Center for Geriatrics, West China Hospital, Sichuan University, Chengdu, China; ^3^The Med-X Center for Manufacturing, Sichuan University, Chengdu, China

**Keywords:** age, brain computer interface, steady state motion visual evoked potential, motion onset visual evoked potential, action observation

## Abstract

**Objective:**

Brain-computer interface (BCI) has great potential in geriatric applications. However, most BCI studies in the literature used data from young population, and dedicated studies investigating the feasibility of BCIs among senior population are scarce. The current study, we analyzed the age-related differences in the transient electroencephalogram (EEG) response used in visual BCIs, i.e., visual evoked potential (VEP)/motion onset VEP (mVEP), and steady state-response, SSVEP/SSMVEP, between the younger group (age ranges from 22 to 30) and senior group (age ranges from 60 to 75).

**Methods:**

The visual stimulations, including flicker, checkerboard, and action observation (AO), were designed with a periodic frequency. Videos of several hand movement, including grasping, dorsiflexion, the thumb opposition, and pinch were utilized to generate the AO stimuli. Eighteen senior and eighteen younger participants were enrolled in the experiments. Spectral-temporal characteristics of induced EEG were compared. Three EEG algorithms, canonical correlation analysis (CCA), task-related component analysis (TRCA), and extended CCA, were utilized to test the performance of the respective BCI systems.

**Results:**

In the transient response analysis, the motion checkerboard and AO stimuli were able to elicit prominent mVEP with a specific P1 peak and N2 valley, and the amplitudes of P1 elicited in the senior group were significantly higher than those in the younger group. In the steady-state analysis, SSVEP/SSMVEP could be clearly elicited in both groups. The CCA accuracies of SSVEPs/SSMVEPs in the senior group were slightly lower than those in the younger group in most cases. With extended CCA, the performance of both groups improved significantly. However, for AO targets, the improvement of the senior group (from 63.1 to 71.9%) was lower than that of the younger group (from 63.6 to 83.6%).

**Conclusion:**

Compared with younger subjects, the amplitudes of P1 elicited by motion onset is significantly higher in the senior group, which might be a potential advantage for seniors if mVEP-based BCIs is used. This study also shows for the first time that AO-based BCI is feasible for the senior population. However, new algorithms for senior subjects, especially in identifying AO targets, are needed.

## Introduction

Brain-computer interface (BCI) is a direct communication pathway between the brain and an external device, receiving increasing attention in aging research and geriatrics. A BCI acquires and analyses brain signals in real time and provides a non-muscular channel of communications and control for individuals with disabilities, majority of which are seniors (Wolpaw et al., 2002). In the BCI research, various independent electroencephalogram (EEG) signals or stimuli-dependent EEG signals have been investigated as the source signal for non-invasive BCI paradigms. Among these, event-related desynchronization/synchronization (ERD/ERS) (Wang et al., 2019) and movement related cortical potentials (MRCP) (Niu and Jiang, 2022) are spontaneous and independent modalities, while P300 (Hoffmann et al., 2008), transient visual evoked potential (VEP) (Liparas et al., 2014), and steady-state visual evoked potential (SSVEP) (Vialatte et al., 2010) are stimuli-dependent. In particular, transient VEPs are elicited by light flashing or patterned stimuli at low rates. SSVEPs are evoked by periodic flickers with a stationary distinct spectrum in EEG recordings mainly at the occipital cortical area. Among the various BCI paradigms, the SSVEP-based BCI has the advantage of a high information transfer rate (ITR) and no or little need for subject training to achieve high decoding accuracy. These advantages make it popular in spelling (Chen et al., 2021) and brain-controlled robots (Chen et al., 2020).

To date, BCI applications have mainly focused on assisting people with disability in participating in daily life activities. Senior individuals accounts for a large proportion of individuals disability. The average age of stroke patients admitted was 66 years old reported in China stroke statistics 2019 (Wang et al., 2020). While there are well-documented age-related changes in EEG signals, few studies in the current BCIs literature investigated the feasibility of various EEG modalities in senior population. For example, researchers have shown that there are changes in the brain with age in processing speed, working memory, inhibitory functions, brain structure size, and white matter integrity (Park and Reuter-Lorenz, 2009). A model which is called hemispheric asymmetry reduction in older adults (HAROLD) stated that older adults tended to be less lateralized than younger adults when performing the same task (Cabeza, 2002). A reduced lateralization of ERD was also reported in the elderly when the participants were performing covert hand movements (Zich et al., 2015). In particular, the algorithms in BCI research are almost exclusively developed with data from young population under the age of 30. The above age-related change has been shown to have a dramatic change on algorithms developed from young subjects’ data when used in senior population (Chen et al., 2019). For example, the reduced lateralization in older subjects caused the accuracy dropping from 82.3 to 66.4% in sensory-motor rhythm (SMR)-based BCI. As such, careful investigations should be conducted before these BCIs algorithms can be applied in geriatric applications.

Steady-state visual evoked potential is one of the most popular non-invasive BCI modalities, and its age-related effect has been investigated. For example, a BCI spelling performance from young adults (between the ages of 19 and 27) and older adults (between the ages of 54 and 76) was compared. The results showed a significant difference in the ITR between these two groups (Volosyak et al., 2017). Another study reported SSVEP-based BCI performance using the medium frequency range (approximately 15 Hz) and the high-frequency range (above 30 Hz) in 86 subjects aged 18–55 (Volosyak et al., 2011). But that study may not be reflective of the general population. The subjects tended to be young men (25.83 ± 7.84 years). And a controlled survey has to be carried in the future to examine the effect of age. In addition, a two-class SSVEP-based BCI was tested in young adults, older adults, and ALS patients. The average accuracy reached 96.1% in young adults, 91.8% in older adults, and 81.2% in ALS patients (Hsu et al., 2016). Thus, age seems to have some influence on the performance of the SSVEP-based BCI.

The flickering stimuli used in SSVEP-based BCI can easily cause visual fatigue and has the risk of eliciting seizures, a potential risk increasing with age. Recently, motion stimuli without flickers have been proposed and attracted more attention. For example, periodic motions, such as rotation, spiral, and radial motion, have been shown to elicit steady-state motion visual evoked potentials (SSMVEPs) (Yan et al., 2018). The SSMVEP-based BCI maintains the low- or no-subject training characteristics of SSVEP-based BCIs, but with the benefit of less visual discomfort (Xie et al., 2012), and higher robustness to competing stimuli (Ravi et al., 2022), among others. Moreover, the perception of motion is one of the fundamental tasks of higher visual systems. The dorsal pathway in the visual system mainly deals with motion, which is a different pathway from that dealing with flicker. Thus, the EEG responses to the motion stimulus is different from that to flicker stimuli. However, to the best knowledge of the authors, no comparison of EEG responses to motion stimuli among different age groups has been reported in the literature.

Steady-state visual evoked potential/steady-state motion visual evoked potential-based BCIs mainly apply to spelling (Chen et al., 2015) and might not directly apply to stroke rehabilitation, which is one of the key sub-group in seniors with disability that BCIs might help. The flicker and the motion stimuli mentioned above can activate the occipital area but have little effect on the sensorimotor area, which is important for stroke rehabilitation purposes (Biasiucci et al., 2018).

The novel action observation (AO)-based BCI shows great potential for promoting motor cortical activation in neural rehabilitation applications. Our recent study showed that observing the carefully designed gaiting stimulus could simultaneously elicit SSMVEPs in the occipital area and induce SMR in the primary sensorimotor area (Zhang et al., 2021b). We have further shown that designed action video of fine hand movements could also simultaneously elicit SSMVEP in the occipital area and SMR in the sensorimotor area (Zhang et al., 2021a). The induced SMR in both cases could be beneficial for motor cortical activation, consequently with great potential in rehabilitation applications. However, the AO-based BCI was only tested in younger subjects, not in senior population.

To evaluate the ability of visual stimuli in BCI, especially the motion stimulus, inducing EEG characteristics in senior subjects, the transient response and the steady state response to three typical paradigms were explored in the current study for the first time. The flicker, motion checkerboard, and AO were selected as the visual stimuli, which were designed with periodic frequency. Not only the steady state response, i.e., SSVEP/SSMVEP, but also the transient response, i.e., VEP/motion onset VEP (mVEP) (Heinrich, 2007), were illustrated in two age groups (young and senior). The amplitudes of the transient components were compared between these two age groups. The feasibility of AO stimuli, such as grasping, the opposition movement, and pinching, eliciting the SSMVEP in senior subjects was also investigated. Furthermore, three typic algorithms, which were widely utilized to identify the target stimulus in the multi-stimuli scenario based on the induced SSVEP/SSMVEP in younger subjects, were investigated to the applicability of dealing with senior EEG signals.

## Materials and methods

### Participants

Two different age groups of healthy volunteer participants, i.e., a senior group and a young group, participated in this study. Each group contained eighteen subjects. The senior group consisted of four males and 14 females, aged 60–75 years old (mean ± SD: 67.50 ± 4.20), and the younger group consisted of nine males and nine females, aged 22 to 30 years old (mean ± SD: 26.17 ± 2.68). All 36 volunteer participants were naïve to BCI.

### Stimulation design

**FIGURE 1:**
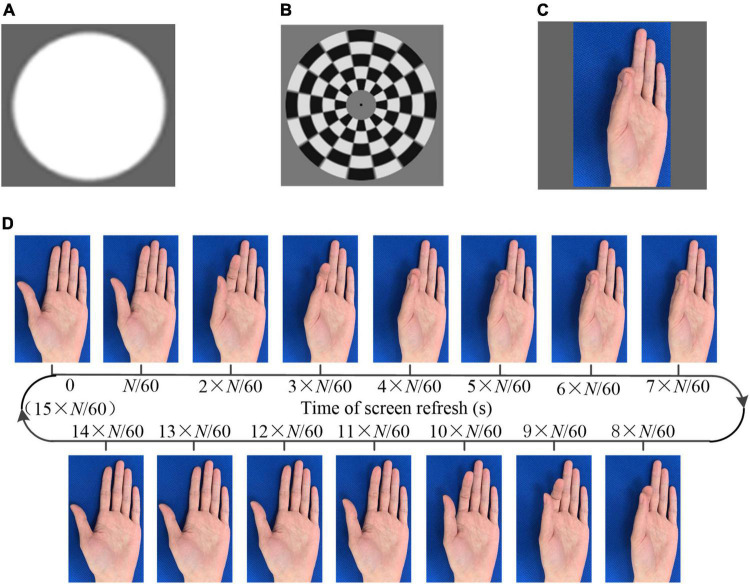
The visual stimulation. **(A)** Flicker **(B)** checkerboard **(C)** action observation **(D)** generation of the action observation (AO) stimulus (pinch).

Three different kinds of visual stimulation, i.e., flicker, checkerboard, and AO, as shown in Figure 1, were designed to induce the visual evoked potential (transient and steady state) in brain. The stimulations were presented on a liquid crystal display monitor. The screen refresh rate was 60 Hz, i.e., 60 frames per second.

The sampled sinusoidal stimulation method (Chen et al., 2014) was utilized to present visual flickers. The period flicker was intended to elicit SSMVEP. Checkerboard stimulation consists of multiple concentric rings (Zhang et al., 2017, 2019). Each ring was divided into white and black lattices with equal sizes and numbers. Thus, the total areas of the bright and dark regions in each ring were always identical. The multiple rings in the checkerboard contracted as the phase of the sinusoid signal of the targeted frequency changed from 0° to 180° and expanded as the phase changed from 180° to 0°. The period movement was intended to elicit SSMVEPs.

In addition, videos of several hand movements, including grasping, dorsiflexion, the thumb opposition, and pinch, were recorded. A frame rate reduction method (Zhang et al., 2021a) was utilized to generate the AO stimulus. Taking the stimulus of pinch as an example, as shown in Figure 1D, each frame was extracted from a video. The same image would last for *N*/60 s, followed by the next different image. Consequently, the frame rate of the designed stimulus decreased to 60/*N* Hz. In one cycle of the action movement, there were a total of *M_AO* captured images. Thus, the frequency of the action movement was 60/(*M_AO* × *N*). The background of the stimuli was unchanged so that the subject intuitive perception was that the finger was moving instead of flicker. If the optical frequency of the stimulus was lower than 4 Hz, the SSVEP cannot be induced. Thus, we designed the AO stimulus containing two frequencies, i.e., the frame rate and the frequency of the action movement. The frame rates were higher than 4 Hz to ensure the effective induction of the SSMVEP. The frequencies of the action movement were approximately 1 Hz, within the normal speed of human movement. In addition to the SSVEP/SSMVEP, the onset of the visual stimulus was anticipated to elicit VEP/mVEP. The program presenting the stimulus was developed with MATLAB using the Psychophysics Toolbox (Brainard, 1997).

### Experimental design

**FIGURE 2:**
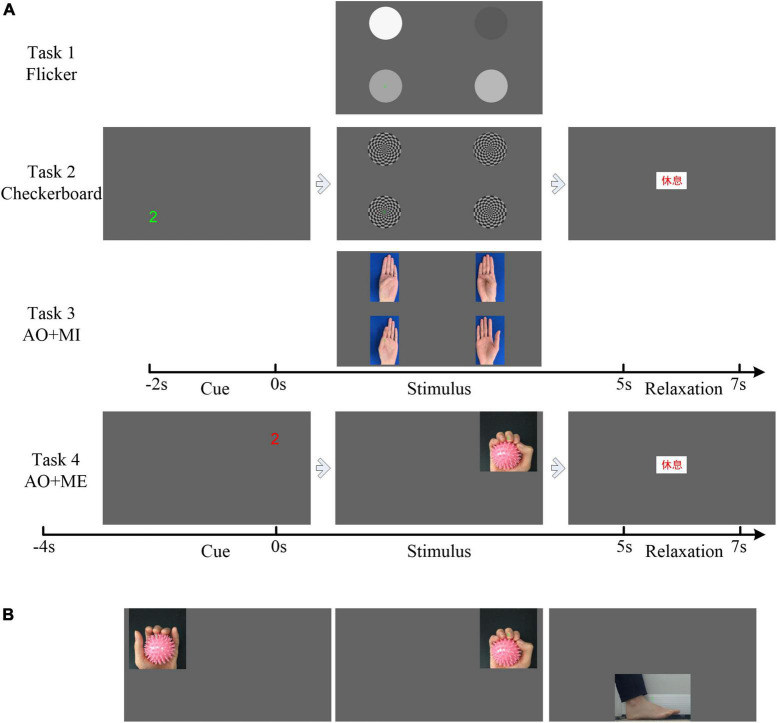
Illustration of the experimental protocol. **(A)** The trial sequence of the four tasks. **(B)** The action observation (AO) stimulus in the stimulus phase used in task 4.

During the experiment, the participant was seated in a comfortable chair and was briefed on the tasks to be performed. In total, four tasks were designed, as shown in Figure 2. Multiple visual stimuli were presented simultaneously in task 1–3. The inter-stimulus distance (ISD) was measured as the visual angles between two individual stimuli. The minimal ISD was 9.46° in the first three tasks. And that ISD had little influence on the decoding performance of SSVEP/SSMVEP-based BCI (Gao et al., 2021). To avoid the unknown effect of competing stimuli which may be a confounding factor for the novel AO stimulus, we further designed task 4 that only one stimulus was presented. Task 1: the subject engaged his or her gaze at the flicker stimulus according to the cues displayed on the screen (Flicker). Task 2: the subject engaged his or her gaze at the checkerboard stimulus (Checkerboard). Task 3: the subject engaged his or her gaze at the AO stimulus and imagined the same movement simultaneously (AO+MI). Task 4: the subject engaged his or her gaze at the AO stimulus and performed the same movement simultaneously (AO+ME). The experimental tasks were designed based on the requirements of the target application, i.e., BCI-based rehabilitation training. In addition to gazing, the participants were instructed to imagine the movement or performance the movement, which will be benefit for rehabilitation training. Thus, the mental tasks were added in the AO stimulus.

Figure 2A illustrates the trial sequence in the experiment. Each trial consisted of three phases: the cue phase, stimulus phase and relaxation phase. For the first three tasks, each trial started with the cue phase (from −2 to 0 s), where one of the four cue letters (“1,” “2,” “3,” and “4”) would appear at the screen, at one corner of the screen. It indicated the target stimulus for the current trial, at which participant would then engage his or her gaze during the stimulus phase. The stimulus phase started at 0 s and lasted 5 s. In this phase, the four stimuli (Task 1: Four flicker stimuli. Task 2: Four cherkerbaord stimuli. Task 3: The left-hand opposition movement stimulus, the left-hand pinch stimulus, the right-hand opposition movement stimulus, and the right-hand pinch stimulus) appeared on the four corners of the screen for 5 s. The flicker frequencies of the four flicker stimuli, the motion frequencies of the four checkerboard stimuli, and the frame rates of the four AO stimuli were all 6, 5, 4.615, and 6.667 Hz. For the AO stimulus, the frequencies of the action movement were 0.6, 0.83, 0.58, and 1.11 Hz, respectively (*M_AO* = 10, 6, 8, and 6, respectively). The participants were asked to gaze at the target appearing in the same position as the letter shown in the cue phase or to imagine the movement based on the task requirement mentioned above. This was followed by the 2 s long relaxation phase, during which the participant could relax the gaze. The text “rest” in Chinese appeared on the screen. The videos of the single trial in each task could be found in the Supplementary material.

The trial sequence in task 4 was similar to that in task 3. The main differences were the cue phase and the stimulus phase. The duration of the cue phase in task 4 was 4 s, and a countdown in 1 s intervals appeared at the screen in the cue phase. In the stimulus phase, only one stimulus would appear in the same position as the countdown shown in the cue phase. Figure 2B shows all possibile AO stimuli (including left-hand grasping, right-hand grasping, and dorsiflexion in the stimulus phase) that could appear in this phase. The frame rates of these three AO stimuli were 10, 6, and 4.615 Hz. The frequencies of the action movement were 1, 1, and 0.77 Hz, respectively (*M_AO* = 10, 6, and 6, respectively).

For the first three tasks, the experiment consisted of four runs in each task. In each run, each of the four targets was repeated five times in a randomized order resulted in a total of 20 experimental trials. Thus, there were a total of 80 experimental trials for each task. For task 4, the experiment consisted of five runs, and each of the three targets was repeated seven times in a randomized order in each run.

### Electroencephalogram data recording

Electroencephalogram signals were recorded using a 32-channel wireless g.Nautilus EEG system (g.tec, Austria). Electrodes were placed at Fp1, Fp2, AF3, AF4, F7, F3, Fz, F4, F8, FC5, FC1, FC2, FC4, T7, C3, Cz, C4, T8, CP5, CP1, CP2, CP6, P7, P3, Pz, P4, P8, PO7, PO3, PO4, PO8, and Oz according to the extended 10/20 system. The reference electrode was located on the right earlobe, and the ground electrode was located on the forehead. A hardware notch filter at 50 Hz was used, and signals were digitally sampled at 500 Hz. All EEG data, event timestamps (the beginning and the end of each trial) and true labels were recorded for subsequent processing.

### Target detection

In this study, canonical correlation analysis (CCA), task-related component analysis (TRCA), and extended CCA-based method (extended CCA) were investigated for target stimulus detection and classification based on SSVEPs/SSMVEPs. The classification accuracy was computed to evaluate the target identification performance in different age groups. The classification accuracy is defined as the percentage of the correct predictions out of all predictions. EEG data from electrodes P3, Pz, P4, PO7, PO3, PO4, PO8, and Oz were selected for analysis. EEG data in the stimulus phase were bandpass filtered from 3 to 40 Hz with the Butterworth filter. Then, the three abovementioned methods were utilized to perform classification. The details of the target identification methods are described below.

### Canonical correlation analysis-based target identification

Canonical correlation analysis is a statistical way to measure the underlying correlation between two multidimensional variables and has been widely used to detect the frequency of SSVEPs (Lin et al., 2006). It is a training free method. Single trial test data are denoted as *X* ∈ ℝ^*N_c_×N_s_*^. Here, *N_c_* is the number of channels, and *N_s_* is the number of sampling points in each trial. In the current study, multichannel EEG data in the occipital region and sine-cosine reference signals were calculated by the following formula.


ρ⁢(x,y)=E⁢[wxT⁢X⁢YfT⁢wy]E⁢[wxT⁢X⁢XT⁢wy⁢E⁢[wxT⁢Yf⁢YfT⁢wy]]

where ρ is the CCA correlation coefficient and *Y_f_* is the reference signal.

The sine-cosine reference signals are as follows:


$$Y_{f}=\left\{\begin{array}{c} \begin{array}{c} \text{sin}\times \left(2\times \pi \times f\times t\right)\\ \text{cos}\times \left(2\times \pi \times f\times t\right) \end{array}\\ \text{sin}\times \left(4\times \pi \times f\times t\right)\\ \text{cos}\times \left(4\times \pi \times f\times t\right) \end{array}\right\}$$

where *f* is the motion frequency.

The target on which the participant focused could be identified by taking the maximum CCA coefficient.

### Task-related component analysis-based target identification

Task-related component analysis is an approach to extract task-related components from a linear weighted sum of multiple time series. TRCA was first proposed to maximize the reproducibility during task periods from near-infrared spectroscopy data (Tanaka et al., 2013). As it had the ability to maximize inter-block covariance and to remove task-unrelated artifacts, TRCA was successfully used as a spatial filter to remove background EEG activities in SSVEP-based BCIs (Nakanishi et al., 2018). The spatial filter can be achieved as follows:


$$\omega =\arg\max_{\omega }\frac{\omega^{T}\,S\,\omega }{\omega^{T}\,Q\,\omega }$$

The normalization matrix Q is defined as:


$$Q=\sum_{j_{1},j_{2}=1}^{N_{c}}C\,o\,v\,\left(x_{j_{1}}\,\left(t\right),x_{j_{2}}\,\left(t\right)\right)$$

where *x_j_*_1_(*t*) is the EEG data in the *j_1_*-th channel and *x_j_*_2_(*t*) is the EEG data in the *j_2_*-th channel. *Cov*(.,.) represents the cross covariance.

The symmetric matrix *S* = (*S_j_*_1_*_j_*_2_)1≤*_j_*_1_,*_j_*_2_≤_*N*_*c*__ is defined as:


$$S_{j_{1}\,j_{2}}=\sum_{\begin{array}{c} h_{1},h_{2}=1\\ h_{1}\neq h_{2} \end{array}}^{N_{t}}C\,o\,v\,\left(x_{j_{1}}^{\left(h_{1}\right)}\,\left(t\right),x_{j_{2}}^{\left(h_{2}\right)}\,\left(t\right)\right)$$

where $x_{j_{1}}^{\left(h_{1}\right)}\,\left(t\right)$ is the EEG signal in the *h_1_*-th trial in the *j_1_*-th channel. $x_{j_{2}}^{\left(h_{2}\right)}\,\left(t\right)$ is the EEG signal in the *h_2_*-th trial in the *j_2_*-th channel.

With the help of the Rayleigh-Ritz theorem, the eigenvector of the matrix *Q*^−1^*S* provides the optimal coefficient vector.

If there were four individual training data corresponding to four stimuli in one task, four different spatial filters could be obtained. Then, an ensemble spatial filter *W* = [ω_1_ω_2_ω_3_ω_4_] was obtained. Through spatial filtering *X^T^W*, the test data *X* were expected to be optimized to achieve maximum performance.

The correlation coefficient was selected as the feature. Pearson’s correlation analysis between the single-trial test signal *X* and averaging multiple training trials as ${\bar{\chi }}_{i}=\frac{1}{N_{t}}\,\sum_{h=1}^{N_{t}}\chi_{i\,h}$ across trials for the *i*-th stimulus was calculated as $r_{i}=\rho \,\left(X^{T}\,W,{\left({\bar{\chi }}_{i}\right)}^{T}\,W\right)$. Here, ρ is Pearson’s correlation, *i* indicates the stimulus index, *h* indicates the index of training trials, and *N_t_* is the number of training trials.

The target on which the participant focused could also be identified by taking the maximum coefficient as *Target* = *max*⁡(*r*_*i*_),*i* = 1,2,3,4.

### Extended canonical correlation analysis-based target identification

The extended CCA-based method incorporated individual template signals obtained by averaging multiple training trials as ${\bar{\chi }}_{i}$ (Chen et al., 2015). The following three weight vectors are utilized as spatial filters to enhance the SNR of SSMVEPs: (1) $W_{X}\,\left(X,{\bar{\chi }}_{i}\right)$ between test signal *X* and individual template ${\bar{\chi }}_{i}$, (2) *W*_*X*_(*X*,*Y*_*f*_) between test signal *X* and the sine-cosine reference signals *Y_f_*, and (3) $W_{{\bar{\chi }}_{i}}\,\left({\bar{\chi }}_{i},Y_{f}\right)$ between the individual template ${\bar{\chi }}_{i}$ and the sine-cosine reference signals *Y_f_*. Then a correlation vector ${\hat{r}}_{i}$ is defined as follows.


r^i=[ρi,1ρi,2ρi,3ρi,4]=[ρ⁢(X,Yf)ρ⁢(XT⁢WX⁢(X,χ¯i),χ¯iT⁢WX⁢(X,χ¯i))ρ⁢(XT⁢WX⁢(X,Yf),χ¯iT⁢WX⁢(X,Yf))ρ⁢(XT⁢Wχ¯i⁢(χ¯i,Yf),χ¯iT⁢Wχ¯i⁢(χ¯i,Yf))]

Finally, the following weighted correlation coefficient is used as the feature in target identification.


ri=∑l=14s⁢i⁢g⁢n⁢(ρi,l)⋅ρi,l2

where *sign*() is used to retain discriminative information from negative correlation coefficients between the test signals and individual templates.

The target on which the participant focused could also be identified by taking the maximum coefficient.

In addition, a fourfold cross-validation scheme was performed for the first three tasks. One run’s data were retained as the validation data for testing, and the other three runs’ data were used as training data. There was no overlapping part in either the training or test subsets. The cross-validation process was then repeated four times, with each of the four runs’ data used exactly once as the validation data. Similarly, a fivefold cross-validation scheme was performed for the fourth task.

### Statistical analysis

A mixed-effect model of ANOVA was used to quantify the differences in the amplitudes of P1 and N2 in each task. The group with two levels (1: senior, 2: young) was one fixed factor. The subject nested within the group and target were two random factors. The mixed-effect model of ANOVA was also used to quantify the differences in the classification accuracies. The method with three levels (1: CCA, 2: TRCA, and 3: extended CCA), data length with three levels (1: 1 s, 2: 3 s, and 3: 5 s), and group with two levels (1: senior and 2: young) were three fixed factors, and subject was a random factor nested within groups.

## Results

### Temporal characteristics of the induced transient response

**FIGURE 3:**
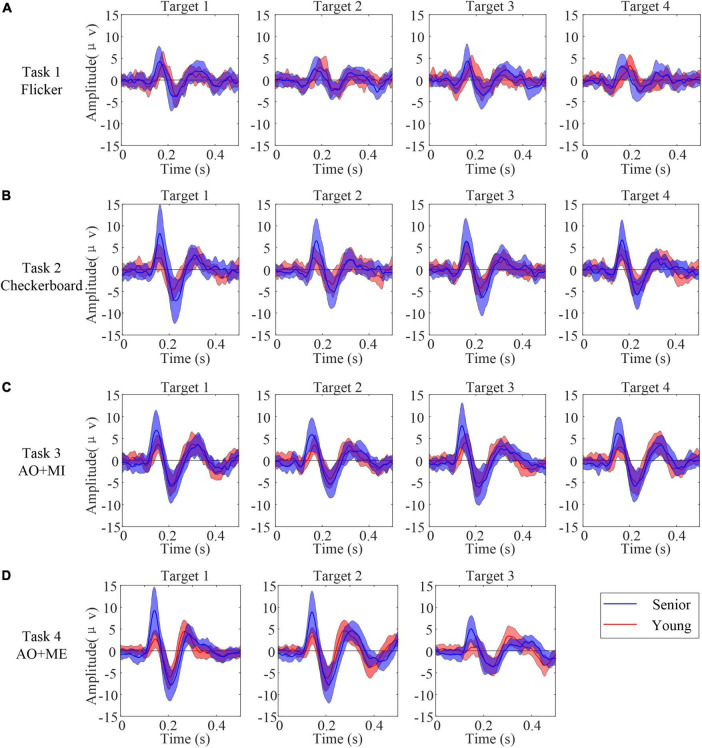
The grand average waveforms for all senior subjects (blue line) and all younger subjects (red line) at electrode PO8 response to different kinds of stimuli. 0 indicate the onset of the stimulus. The center dashed line shows the averaged amplitudes while the shading around the dash line indicates averaged amplitudes ± SD. **(A)** Task 1 **(B)** task 2 **(C)** task 3 **(D)** task 4.

High contrast and bright luminance of a visual object was able to evoke VEP, and the motion behavior of visual objects could evoke mVEP. Thus, the transient EEG components immediately following the onset of the visual stimuli were first analyzed. EEG data epochs during the beginning of the stimulus phase (0–0.5 s) were averaged over all trials registered from each of the subjects. Figure 3 illustrates the grand average waveforms for all senior subjects (blue line) and all younger subjects (red line) at electrode PO8 response to different kinds of stimuli. 0 s referred to the moment when the stimulus occurred on the screen. Overall, the grand average visually evoked potentials in response to target stimuli had a positive deflection (P1) with a latency of 100–160 ms, followed by a negative deflection (N2) with a latency of 160–230 ms post-flicker or postmotion onset. The transient response to the motion stimulus was clearer than that to the flicker. Furthermore, the amplitudes of the P1 and N2 repsonses to the flicker stimulus were similar in these two age groups. But the amplitudes of the P1 and N2 repsonses to the motion stimulus in the senior group were larger than those in the younger group. For example, in the AO+MI task, the amplitudes (mean ± SD) of those two components were 7.70 ± 4.03 μV and −7.01 ± 3.34 μV in senior group, whereas the amplitudes (mean ± sd) of P1 and N2 were 4.69 ± 1.85 μV and −6.01 ± 2.35 μV in younger group.

A mixed-effect model of ANOVA was used to quantify the differences in the amplitudes of P1 and N2 in different tasks. In task 1 (Flicker), the fix factor Group had no significant influences on the amplitudes of P1 [*F*_(1,__34)_ = 3.71, *p* = 0.063] and N2 [*F*_(1,__34)_ = 0.71, *p* = 0.405]. The random factor Target also had no significant influence on the amplitude of the P1 (*p* = 0.126) and N2 (*p* = 0.127). In task 2 (Checkerboard), the mixed-effect model of ANOVA revealed that the fix factor Group had a significant influence on the amplitudes of the P1 [*F*_(1,__32.18)_ = 12.12, *p* = 0.001]. But the Group had no significant influence on the amplitude of N2 [*F*_(1,__34)_ = 3.78, *p* = 0.06]. The random factor Target had no significant influence on the amplitude of P1 and N2 responses to the checkerboard stimulus (*p* = 0.205 and 0.132, respectively). For the AO stimulus in task 3 and 4, the mixed-effect model of ANOVA revealed that the fix factor Group had a significant influence on the amplitudes of the P1 in task 3 [*F*_(1,__34)_ = 10.1, *p* = 0.003] and in task 4 [*F*_(1, 6.98)_ = 11.82, *p* = 0.011]. But the Group had no significant influence on the amplitude of N2 in task 3 [*F*_(1, 31.97)_ = 1.31, *p* = 0.216] and in task 4 [*F*_(1, 7.81)_ = 1.7, *p* = 0.229]. The random factor Target had no significant influence on the amplitude of P1 and N2 in task 3 (*p* = 0.161 and 0.137, respectively) and in task 4 (*p* = 0.276 and 0.19, respectively).

**FIGURE 4:**
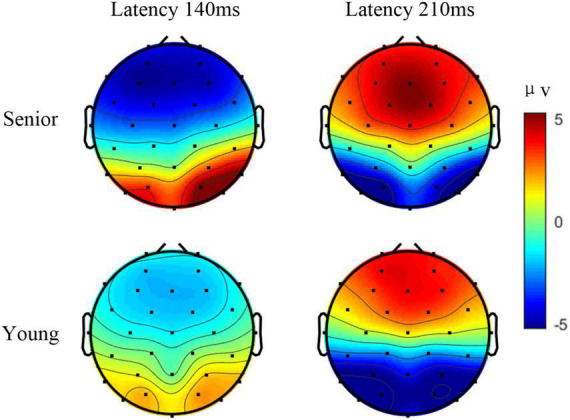
The grand average amplitude topographic maps of target 1 in task 3 among all the senior subjects and all the younger subjects at 140 and 210 ms.

In addition, the type of motion (translation, contraction, expansion, and rotation) might differentially affect the spatial and temporal characteristics of mVEP (Delon-Martin et al., 2006). For example, the N2 component response to the moving line (moving leftward) had an asymmetrical activation in occipito-temporal area (Wang et al., 2006). The P1 and N2 components that respond to expansion and contraction, such as Newton’s ring stimulus or the checkerboard stimulus, exhibited symmetrical activations in the occipital lobe (Xie et al., 2012). Thus, as the AO stimulus was a new paradigm, the topographies for P1 and N2 in task 3 were further analyzed. Figure 4 illustrates the grand average amplitude topographic maps of target 1 among all the senior subjects and all the younger subjects at 140 ms and 210 ms. In both age groups, the topographies for P1 and N2 were all nearly symmetric.

### Spectral characteristics of the induced steady state response

**FIGURE 5:**
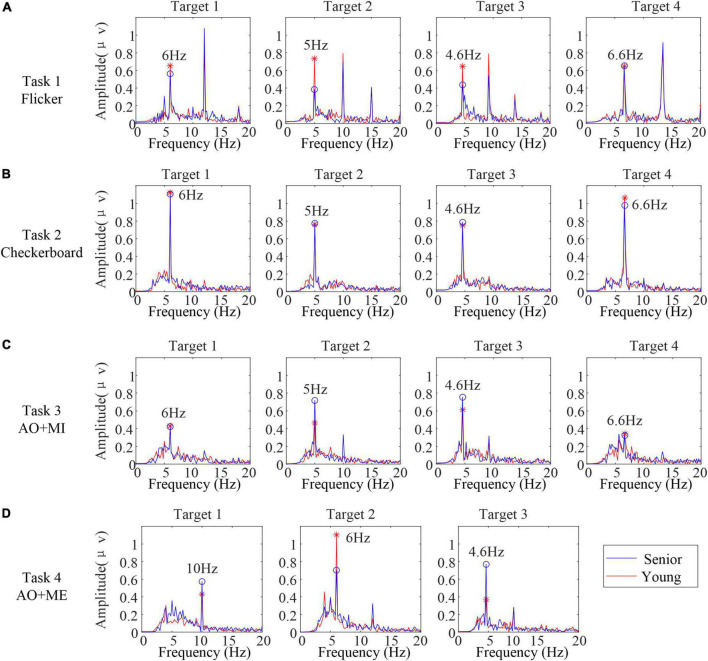
The spectra of electroencephalogram (EEG) data averaged from all the trials and all the subjects in the same age group from Oz in different tasks. **(A)** Task 1 **(B)** task 2 **(C)** task 3 **(D)** task 4.

In addition to the above induced transient response, the steady-state rhythmic EEG components in different age groups were explored. The steady-state response is mainly reflected in frequency domain. Thus, the spectral characteristics of SSVEP/SSMVEP induced by the stimuli in different age groups were analyzed. The spectra of the EEG data in the stimulus phase (0–5 s) were calculated. Figure 5 illustrates the spectra of EEG data averaged from all trials and all subjects in the same age group (blue line: senior and red line: young) from Oz in different tasks. Overall, for all stimulus frequencies, a clean peak at the stimulus frequency in each spectrum was clearly situated at the corresponding frequency when subjects in both age groups stared at each target. The second harmonic of the stimulus frequencies were either unexpectedly weak or completely absent in SSMVEPs induced by the checkerboard, which was different from the SSVEPs induced by the flicker. For the AO stimulus, the second harmonic of the stimulus frequencies occurred in some targets.

Furthermore, for the SSVEPs induced by the flicker, the averaged amplitudes of the peak at the flicker frequencies in senior subjects were slightly lower than those in younger subjects. For the SSMVEP induced by the checkerboard, the averaged amplitudes of the peak at the motion frequencies between senior subjects and younger subjects were highly similar. For the SSMVEP induced by the AO stimulus, the averaged amplitudes of the peak at the frame rates in these two age groups were different among different targets.

### Classification accuracies

**FIGURE 6:**
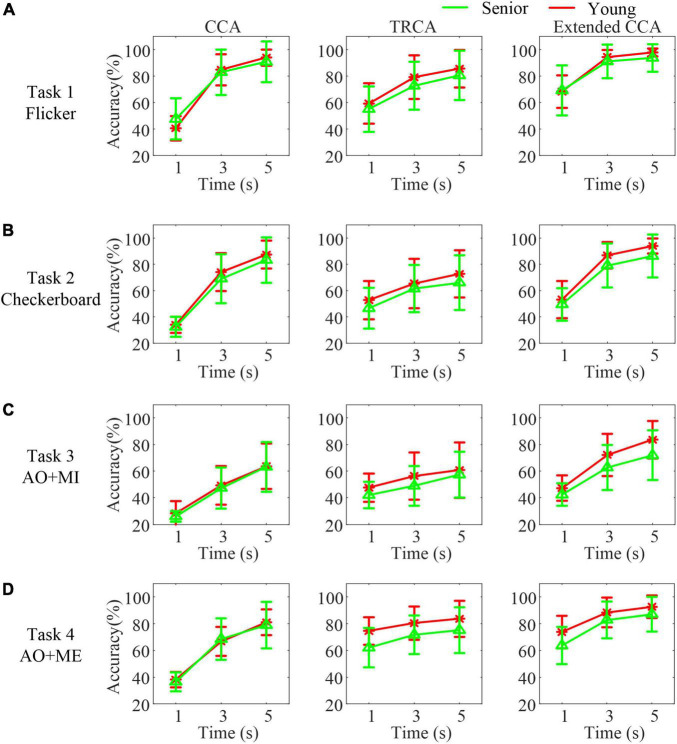
The classification accuracies with different data lengths in different age groups in different tasks. **(A)** Task 1 **(B)** task 2 **(C)** task 3 **(D)** task 4.

To compare the BCI system performance between senior subjects and younger subjects, three algorithms were utilized to detect SSVEPs/SSMVEPs and classify the target stimulus. Figure 6 illustrates the classification accuracies with different data lengths in different age groups (green line: senior and red line: young). As expected, with the increase in the EEG epoch length, the classification accuracies increased monotonically in both groups, regardless of the algorithm used. The average accuracies in the senior group were lower than those in the younger group utilizing the CCA-based method, TRCA-based method and extended CCA-based method in most cases.

A mixed-effect model of ANOVA was used to quantify the differences in the accuracies in each task. The analysis results showed that the fix factors method and data length both had a significant effect on the accuracies (all *p* < 0.001) in all tasks, as expected. However, the fix factor group had no significant effect on the accuracies in all tasks [task 1: *F*_(1,__34)_ = 0.34, *p* = 0.564, task 2: *F*_(1,__34)_ = 1.6, *p* = 0.214, task 3: *F*_(1,__34)_ = 1.73, *p* = 0.198, and task 4: *F*_(1,__34)_ = 2.58, *p* = 0.117].

In addition, compared with the CCA-based method, the TRCA-based method mainly achieved superior performance with a 1 s data length in both groups. The extended CCA-based method achieved the best identification performance in all data lengths in both groups compared with the other two methods. Moreover, the accuracy of identifying the traditional flicker stimulus was highest compared with other stimuli in both groups. The average accuracy achieved 93.75 ± 10.33% within 5 s data length in the senior group. And the average accuracy achieved 97.85 ± 2.93% within 5 s data length in the younger group. The accuracy of identifying the motion checkerboard stimulus was slightly lower. The average accuracies achieved were 86.39 ± 16.37% in the senior group and 93.96 ± 5.68% in the younger group. The accuracy of identifying the AO stimulus in task 3 was lowest compared with other stimuli. The average accuracies were 71.88 ± 18.68% in the senior group and 83.61 ± 14.08% in the younger group. While the average accuracies of identifying the AO stimulus in task 4 (three targets) achieved 87.09 ± 12.87% in the senior group and 92.65 ± 8.47% in the younger group.

Furthermore, the difference in the accuracies utilizing the CCA-based method between these two age groups was similar. However, differences in the accuracies utilizing the TRCA-based method or extended CCA-based method were great, especially when identifying AO stimuli. For example, in task 3, the average accuracy utilizing the extended CCA-based method increased 20% compared with the CCA-based method with 5 s data length in the younger group. However, in the senior group, the accuracies utilizing the extended CCA-based method only increased 8.8% compared with those utilizing the CCA-based method. A pairwise *t*-test revealed that the senior group had a significantly larger difference in the accuracies, utilizing the extended CCA-based method and the accuracies utilizing the CCA-based method with 5 s data length, than the younger group (*t* = −3.35, *p* = 0.004) in task 3.

**FIGURE 7:**
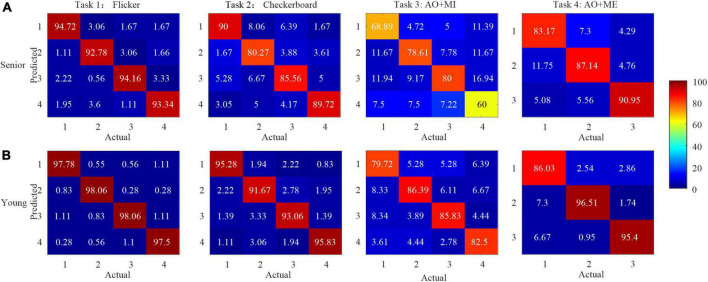
The confusion matrices of the identification accuracy (%) utilizing extended canonical correlation analysis (CCA)-based method with 5 s data length for all participants in the same age group in different tasks. **(A)** Senior group **(B)** younger group.

To further compare the relative identification performance of the stimulus targets based on the multichannel classification methods in different age groups, the confusion matrices of the identification accuracy utilizing the extended CCA-based method with 5 s data length for all participants in the same age group were calculated, as shown in Figure 7. The color scale revealed the average classification accuracies and the diagonals labeled the correct classification accuracies among all the participants. We observed that the influence of the four stimulus frequencies on the accuracies was not too great. We also observed that the identification accuracy of the target 4 was lower than other targets in task 3, which might be caused of the unexpected peak in the spectrum and inducing the low amplitude at the stimulus frequency in target 4 as shown in Figure 5C.

## Discussion and conclusion

The results of the current study indicate that (1) the motion checkerboard and AO stimuli are able to elicit prominent mVEP with specific P1 peak and N2 valley. The amplitude of the P1 peak in the senior group was significantly higher than that in the younger group, which might be a potential advantage for seniors in leveraging mVEP detection to identify when the user engages with a visual stimulus. (2) Except for the traditional flicker and the checkerboard stimulus with periodic frequency, the newly designed AO stimulus can also elicit clearly SSMVEPs at the stimulus frequency in the senior group. As such, the SSVEP/SSMVEP-based BCI is feasible for the senior population. And with the AO stimuli, it has great potential for rehabilitation applications, common in senior population.

To the best of our knowledge, this is the first study to report the differences in both transient response and steady-state response to different kinds of visual stimuli between younger subjects and senior subjects. In the literature, one study utilized the approach of capturing the EEG segment occurring at the onset of the SSVEP stimulus to analyze the transient VEP (Xie et al., 2012). And the spectral-temporal characteristics of the EEG induced by the checkerboard are similar to the results in that study, which utilized the oscillating Newton’s rings as the motion stimuli with young subjects.

In general, low stimulus frequencies (lower than 4 to 6 Hz) could evoke a transient VEP. As the stimulus frequencies increase, the EEG responses overlap one another and merge into the steady state. Thus, transient VEP only occurs at the stimulus onset, and then the EEG response changes into SSVEPs/SSMVEPs in each trial in the current study. In contrast with flash VEP, mVEP has a much lower amplitude decrease with increasing retinal eccentricity (Schlykowa et al., 1993). mVEP displays the largest amplitudes and the lowest inter- and intra-subject variabilities (Guo et al., 2008). This might be the reason that the motion stimulus elicits strong mVEP, while the transient VEP elicited by the flicker was weak in the current study.

Interestingly, the significantly larger amplitude in P1 in mVEP in the senior group was unexpected. One possible reason is that the senior subjects paid more attention than younger subjects, when either checkerboard or AO stimulus was presented. Many senior subjects reported, post-experiment, that they were involuntarily counting the times of the movement in the stimulus during the experiments, while no participants in the younger group reported doing so. Previous studies found that the amplitudes of mVEP components can be modulated by attention (Torriente et al., 1999). “Counting” may help subjects to be more engaged and concentrate more on the target stimulus. In the literature, larger amplitudes of VEP in older females, compared with younger females, were reported, in which the authors suggested that this was due to a heightened sensitivity of the older female visual system to patterned stimuli (La Marche et al., 1986). Furthermore, the characteristics of mVEP are influenced by the type of visual stimuli. The reason for the larger amplitude in mVEP still needs further investigation. The characteristics of the mVEP in senior subjects may provide a valuable signal modality in designing more appropriate BCIs for targeting the senior population. This will be the focus of our future studies. Moreover, the HAROLD model is mainly supported by evidence in the domains of episodic memory, semantic memory, working memory, perception, and inhibitory control. For the EEG response to the visual stimulus, it exhibited symmetrical activations in the occipital lobe as shown in Figure 4. The HAROLD model seems to be not applicable.

On the other hand, the steady-state response, i.e., SSVEP/SSMVEP, provides an even more robust way of determining the subject’s choice of visual target. While no significant difference was found, the average accuracy of identifying the flicker stimulus achieved 93.75% in senior subjects, which was lower than the accuracy in younger subjects (97.85%), with no statistical significance. A similar level of difference in accuracies in these two age groups was also reported a prior study (Volosyak et al., 2017). The current study also reported that no significant difference was found in the accuracies of identifying the checkerboard stimulus or the AO stimulus in the two age groups despite no previous reports. Consistent with this lack of difference, the BCI performance between the two groups were not significant. This might be because the EEG data from channels O1, O2, and POz were not utilized in the classification. Limited by the EEG device, where the positions of the electrodes are fixed and no electrodes were located at O1, O2, and POz.

In the current study, three popular SSVEP algorithms were investigated. In recent literature, many studies have utilized TRCA-based methods to process SSVEPs with short durations (<1 s) and have reported enhanced performance (Nakanishi et al., 2018). The current study also achieved improved accuracy with 1 s data length compared with the more traditional CCA-based method. However, for longer data lengths, the current study found no difference in performance between CCA-based and TRCA-based methods. Furthermore, the extended CCA-based method achieved the highest accuracy in three data lengths.

**FIGURE 8:**
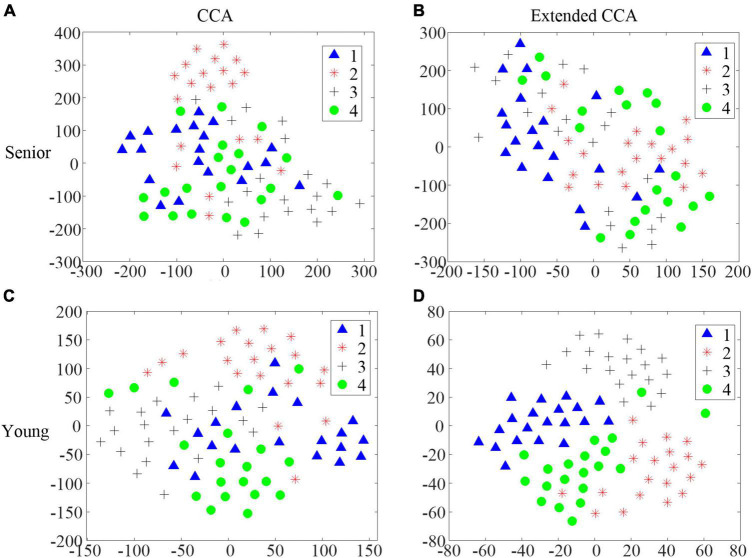
The t-distributed stochastic neighbor embedding (t-SNE) of the correlation coefficients in different age groups. **(A)** The distribution of the CCA correlation coefficient ρ in S9 in the senior group. **(B)** The distribution of the weighted correlation coefficient *r* in S9 in the senior group. **(C)** The distribution of the CCA correlation coefficient ρ in S9 in the younger group. **(D)** The distribution of the weighted correlation coefficient *r* in S9 in the younger group.

It is worth noting that the improvement in accuracy, utilizing the extended CCA-based method compared with the CCA-based method, in the senior group was significantly lower than that in the younger group for the AO stimulus in task 3. Even though the accuracies utilizing the CCA-based method were similar between these two age groups, the distributions of the CCA correlation coefficient were quite different. T-distributed stochastic neighbor embedding (t-SNE) (Van Der Maaten and Hinton, 2008) was utilized to visualize the distribution of the CCA correlation coefficient ρ and the weighted correlation coefficient *r* in different groups. Figure 8 illustrates the t-SNE of S9 in the senior group and S9 in the younger group. The classification accuracy was 70% utilizing the CCA-based method in S9 in the senior group. S9 in the younger group achieved slightly lower accuracy, i.e., 68.75%. The target on which the participant focused was identified by taking the maximum CCA coefficient. However, the boundaries among the four categories are clearer for the younger group (Figure 8C), than in the senior group (Figure 8A). This implies that the quality of the individual training data to build the CCA-based spatial filter, as distributed in the extended CCA-based method, is better in the younger group. Thus, the extended CCA-based method achieved better performance improvement in the younger group, which could also be reflected in Figures 8B,D. Thus, detecting SSMVEPs induced by AO stimuli for seniors calls for better more complex algorithms, which is another research direction.

Furthermore, the current study shows for the first time that the AO-based BCI is feasible for the senior population. The average accuracies of identifying the target stimulus on which the senior participant focused among four AO stimuli (i.e., the left-hand opposition movement, the left-hand pinch, the right-hand opposition movement, and the right-hand pinch) and three AO stimuli (i.e., left-hand grasping, right-hand grasping, and dorsiflexion) reached 71.88 ± 18.68% and 87.09 ± 12.87%, respectively. It is observed that the accuracies in these two tasks are quite different. Even though the stimulations are both AO stimuli in task 3 and task 4, several differences exist. First, it is the four-target classification in task 3, while it is the three-target classification in task 4. In addition, the parameters of the AO stimulus, such as different frame rates and different actions, are different. This finding is in line with our recent study, which showed that the parameters of the AO stimulus influence the classification accuracy (Zhang et al., 2021a).

Overall, the current study reports the differences in spectral-temporal characteristics of the EEG induced by the three types of visual stimuli: flicker, checkerboard, and AO, in both senior and younger groups. The characteristics of the mVEP in senior subjects may provide a valuable signal modality in designing more appropriate BCIs for targeting the senior population. Not only the traditional flicker and the checkerboard stimulus with periodic frequency but also the newly designed AO stimulus, including various actions, could clearly elicit SSMVEP at the stimulus frequency in both age groups. The accuracies of identifying SSVEP/SSMVEP in the senior group were slightly lower than those in the younger group. These findings may be helpful for researchers designing algorithms to achieve high classification performance, specifically for senior subjects. In addition, current study focused on the transient and steady state responses in occipital cortex to different visual stimuli in senior and younger subjects and investigating the applicability of typical algorithms in dealing with senior EEG signals. Analysis of the SMR and the brain network in task 3 and 4 will be our next study.

## Data availability statement

The raw data supporting the conclusions of this article will be made available by the authors, without undue reservation.

## Ethics statement

The studies involving human participants were reviewed and approved by the Ethical Committee of West China Hospital of Sichuan University. The patients/participants provided their written informed consent to participate in this study.

## Author contributions

XZ drafted the manuscript and analyzed the EEG data. NJ and WH provided the suggestion of experiment design. XZ and YJ collected the EEG data. NJ revised the manuscript. All authors contributed to the article and approved the submitted version.
